# Medical radiation exposure in inflammatory bowel disease: an updated meta-analysis

**DOI:** 10.1186/s12876-024-03264-1

**Published:** 2024-05-18

**Authors:** Chao Lu, Xin Yao, Mosang Yu, Xinjue He

**Affiliations:** Department of Gastroenterology, The First Affiliated Hospital, Zhejiang University School of Medicine, Hangzhou, 310003 China

**Keywords:** Radiation exposure, Inflammatory bowel disease, Crohn’s disease, Ulcerative colitis

## Abstract

**Background:**

There have been previous studies and earlier systematic review on the relationship between inflammatory bowel disease (IBD) and radiation exposure. With the diversification of current test methods, this study intended to conduct a meta-analysis to evaluate the IBD radiation exposure in recent years.

**Methods:**

Three databases (PUBMED, EMBASE, and MEDICINE) for relevant literature up to May 1, 2023 were searched. The statistical data meeting requirements were collated and extracted.

**Results:**

20 papers were enrolled. The overall high radiation exposure rate was 15% (95% CI = [12%, 19%]) for CD and 5% (95% CI = [3%, 7%]) for UC. The pooled result found that high radiation exposure rate was 3.44 times higher in CD than in UC (OR = 3.44, 95% CI = [2.35, 5.02]). Moreover, the average radiation exposure level in CD was 12.77 mSv higher than that in UC (WMD = 12.77, 95% CI = [9.93, 15.62] mSv). Furthermore, radiation exposure level of CD after 2012 was higher than those before 2012 (26.42 ± 39.61vs. 23.76 ± 38.46 mSv, *P* = 0.016), while UC did not show similar result (11.99 ± 27.66 vs. 10.01 ± 30.76 mSv, *P* = 0.1). Through subgroup analysis, it was found that disease duration (WMD = 2.75, 95% CI = [0.10, 5.40] mSv), complications (OR = 5.09, 95% CI = [1.50, 17.29]), and surgical history (OR = 5.46, 95% CI = [1.51, 19.69]) significantly increased the proportion of high radiation exposure.

**Conclusion:**

This study found that radiation exposure level of IBD patients was high, which revealed the radiation risk in the process of diagnosis and treatment of IBD patients. In the future, longer follow-up and prospective studies are needed to reveal the relationship between high radiation exposure and solid tumorigenesis.

**Supplementary Information:**

The online version contains supplementary material available at 10.1186/s12876-024-03264-1.

## Background

Inflammatory bowel disease (IBD), including Crohn’s disease (CD) and ulcerative colitis (UC), refers to a group of lifelong idiopathic disorders characterized by gastrointestinal inflammation and extra-intestinal manifestations [1]. The incidence and prevalence of IBD is increasing worldwide especially in Asia, while it is still highest among developed countries in Europe and America [2]. Due to the unclear pathogenesis and the complexity of treatment, IBD has a significant disease and economic burden [3]. The diagnosis of IBD is a difficult and complicated process. In addition to gastrointestinal endoscopy, repeated imaging tests are also required, especially for the diagnosis of CD, which needs to assess the extent and severity of the disease, and the presence of complications [4]. Therefore, the assessment of radiation exposure is very important.

IBD itself increases the risk of intestinal tumors [5, 6], and the use of drugs such as azathioprine, other immunosuppressive agents and biological agents will increase the risk of malignant tumors such as lymphoma [7]. In addition, exposure to ionizing radiation may potentially increase the risk of malignancy [8]. Radiation exposure as low as 50 millisieverts (mSv) has been associated with the development of certain solid tumors such as colon, bladder cancer [9]. Globally, up to 2% of malignancies can be attributed to diagnostic medical radiation (DMR) [10]. Although some clinicians believe that DMR exposure is indeed a potential risk, the actual exposure of IBD patients in clinical practice still lacks sufficient multicenter large sample data to support, that leads to many concerns for patients, such as whether they are exposed to excessive DMR. Previous meta-analysis study have shown that IBD patients do have higher DMR [11]. With the continuous development of medical technology, such as the application of MRI and intestinal ultrasound, it is not clear whether DMR has changed from before.

Therefore, it is important to conduct this study to update our current knowledge by meta-analysis to analyze relevant studies found to date, especially recent studies, to determine the pooled prevalence of increased exposure in IBD patients and risk factors associated with exposure to potentially harmful ionizing levels.

## Methods

### Data selection

We searched three databases (PUBMED, EMBASE, and MEDICINE) for relevant literature up to May 1, 2023. Literature search limited to human studies and English version, including prospective and retrospective studies. The following search terms were used to retrieve potential articles: ((Inflammatory Bowel Disease) OR (IBD) OR (Crohn’s disease) OR (CD) OR (ulcerative colitis) OR (UC)) AND ((radiation exposure) OR (radiation injuries) OR (medical radiation)).

The search was independently performed by 2 authors according to title and abstract, and full text was retrieved if it met the requirement. In addition, disagreement would be evaluated by a third author independently.

### Inclusion criteria and quality assessment

The diagnosis of IBD was based on symptoms, imaging, and histopathology [12]. High diagnostic medical radiation exposure was defined as ≥ 50 mSv. In addition, sufficient data for calculation were needed for inclusion in the study. STROBE checklist was used to assess Quality assessment and risk of bias for the studies included [13]. Moreover, the work was conducted in accordance with the Preferred Reporting Items for Systematic Reviews and Meta-Analyses (PRISMA) statement [14].

### Data extraction

Relevant data from every included study according to the unified standard were extracted by two independent authors and then they proceeded to cross-check the results. The extracted data contained author, country or region, published year, number of subjects, radiation exposure dose, number of high diagnostic medical radiation exposure and factors affecting radiation exposure. Agreement between the investigators was greater than 95%, and differences between the datasets were resolved by discussion.

### Statistical analysis

Continuous variables were expressed as mean and standard deviation, and dichotomous variables were described by odds ratio (OR) and 95% confidence interval (CI). Heterogeneity of the data was quantified with the I^2^ statistic and assessed by Cochran’s Q statistic. In this study, when heterogeneity was less than 50%, the pooled estimates were obtained using the fixed-model (Mantel and Haenszel) method. On the contrary, the random-model (M-H heterology) method was chosen if heterogeneity was more than 50% [15]. This study compared the following: difference in radiation exposure between CD and UC; difference in high diagnostic medical radiation exposure between CD and UC; difference in radiation exposure of CD and UC patients before and after 2012 (According to the articles, it can be basically determined that articles after 2012 did not overlap the count of CT before), and the difference in radiation exposure under different influencing factors including disease duration, gender, complications, surgical history and medication. In addition, sensitivity analysis was used to evaluate whether the results were reliable. Begg’s test was conducted to estimate publication bias with a value of *P* > 0.05 suggesting no publication bias. All data analysis methods involved in this study were implemented through STATA 15 (StataCorp., College Station, Tex, USA).

## Results

### Basic characteristics

**Fig. 1:**
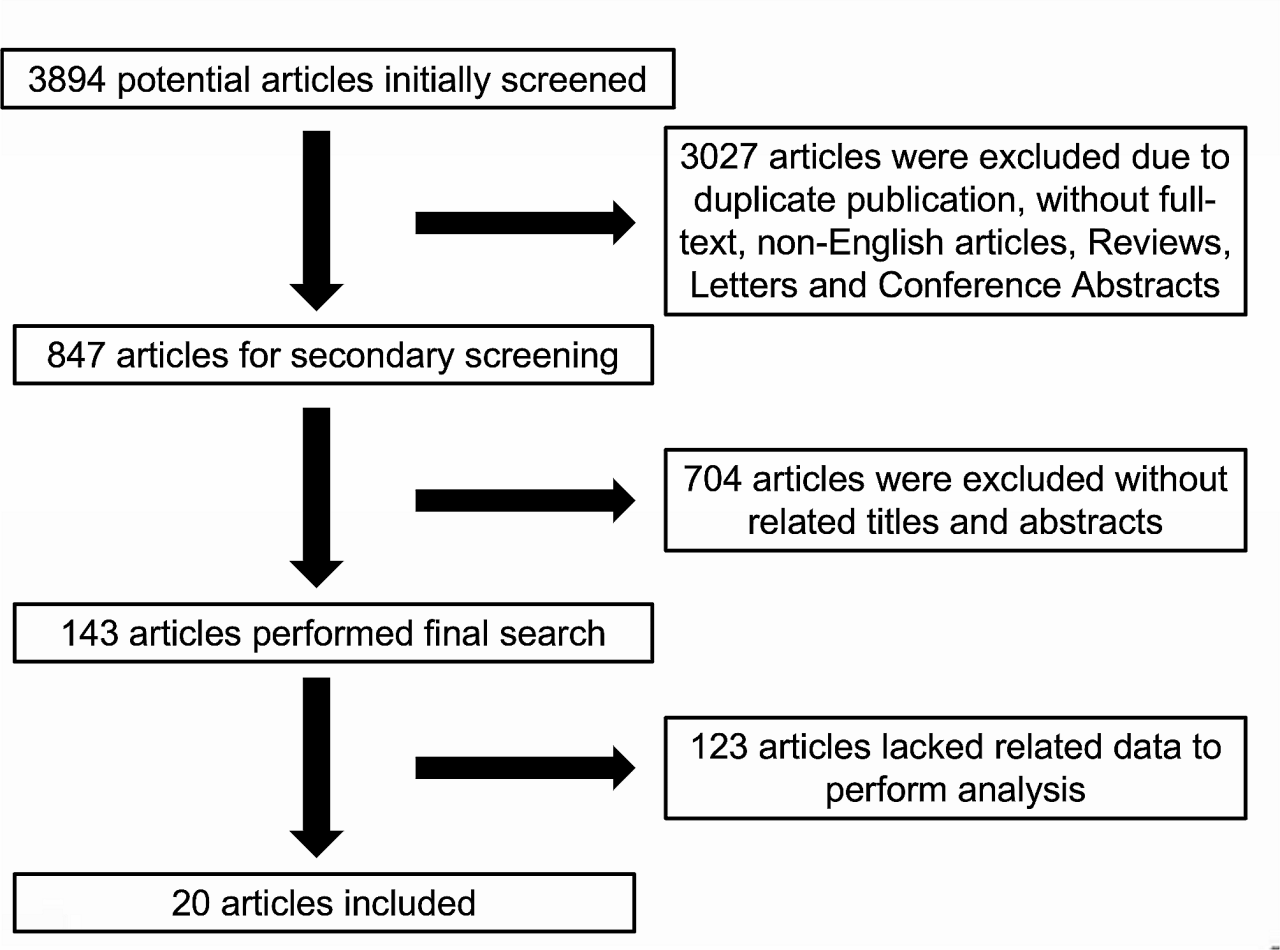
Flowchart of articles selected

A total of 3894 relevant articles were screened, of which 20 papers were enrolled finally according to inclusion criteria. The flowchart has been schematically outlined in Fig. 1 which described the process of the study selection. 20 articles all referred to CD, and 15 of them referred to radiation exposure of UC. The included population of 17 articles came from Europe and the United States. Of the 20 articles reporting on CD, 17 mentioned average radiation exposure values, 16 mentioned numbers of high diagnostic medical radiation exposure, and 13 articles were published after 2012. Of the 15 articles reporting on UC, 13 mentioned average radiation exposure values, 12 mentioned numbers of high diagnostic medical radiation exposure, and 9 articles were published after 2012. UC did not perform subgroup analysis on influencing factors due to lack of literature support. About CD, 3 referred to disease duration, 3 referred to gender, 3 referred to complications, 4 referred to surgical history, and 3 referred to medication.

### Radiation exposure in CD and UC patients

**Fig. 2:**
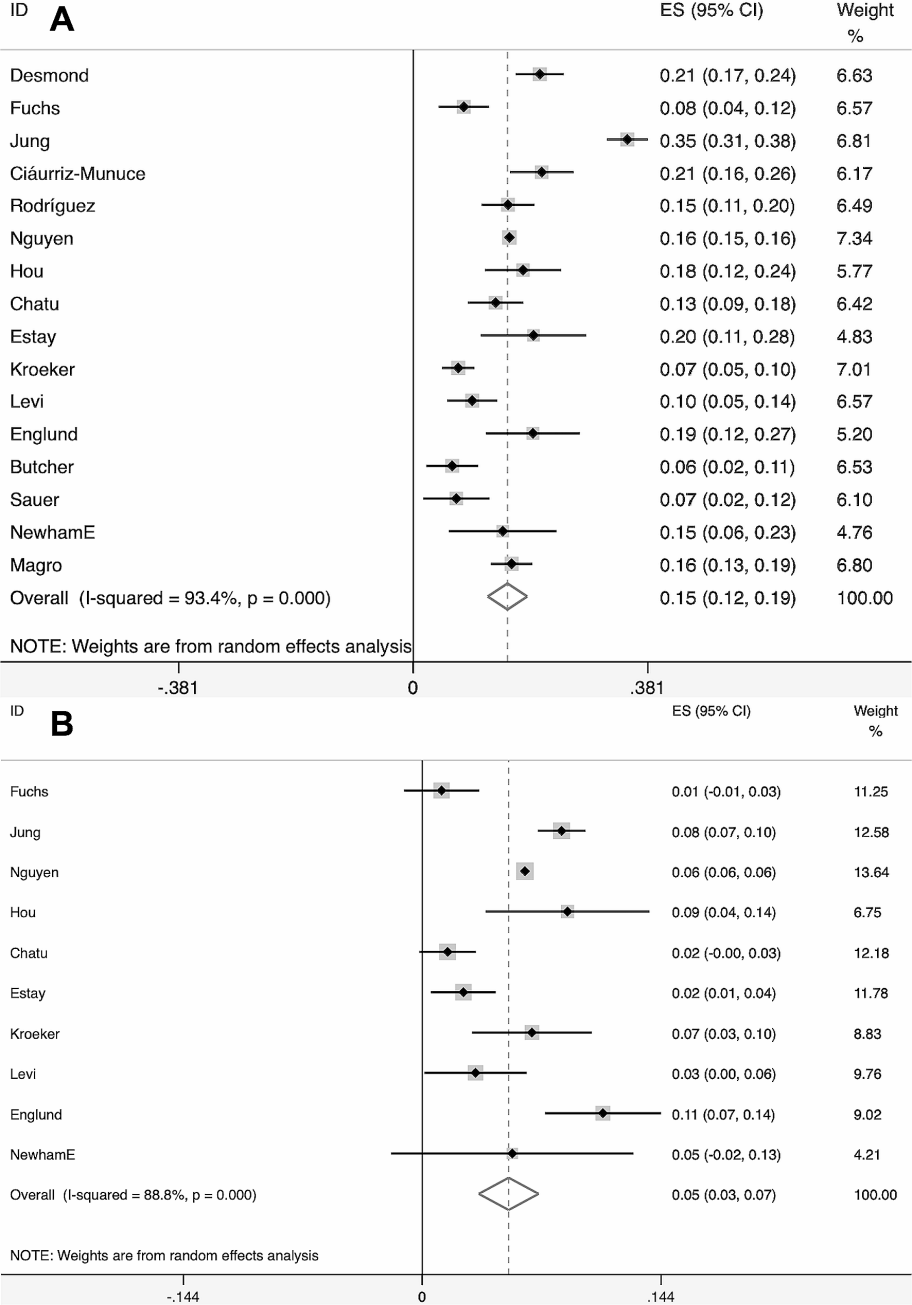
Forest plot showed event rate defined as proportion of patients exposed to high diagnostic medical radiation exposure ≥ 50 mSv in CD and UC patients

**Fig. 3:**
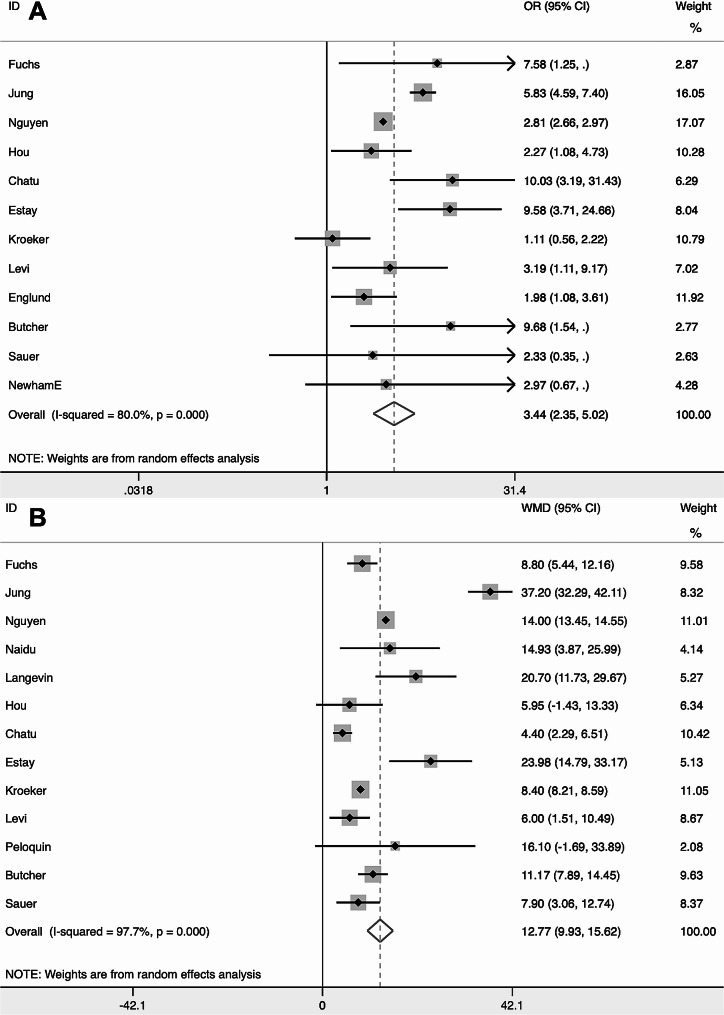
Forest plot showed the difference between radiation exposure level and high radiation exposure odds ratios between CD and UC

The total number and number of individuals with high radiation exposure of CD was 32,963 and 5181 respectively, and the average radiation exposure level was 26.31 mSv (Table 1). At the same time, The total number and number of individuals with high radiation exposure of UC was 34,854 and 2147 respectively, and the average radiation exposure level was 11.97 mSv (Table 2). Combining rates by meta-analysis found that the overall high radiation exposure rate was 15% (95% CI = [12%, 19%]) for CD (Fig. 2A) and 5% (95% CI = [3%, 7%]) for UC (Fig. 2B). The pooled result of meta-analysis found that high radiation exposure rate was 3.44 times higher in CD than in UC (OR = 3.44, 95% CI = [2.35, 5.02]) (Fig. 3A). Moreover, the pooled results of meta-analysis showed that the average radiation exposure level in CD was 12.77 mSv higher than that in UC (WMD = 12.77, 95% CI = [9.93, 15.62] mSv) (Fig. 3B).

**Table 1:** Characteristics of radiation exposure in CD patients

Author	Country	Year	Numbers	Radiation exposure (mSv)	High radiation exposure(*n*)
NewhamE	Australia	2007	62	-	9
Peloquin	USA	2008	103	26.6 ± 69.75	-
Desmond	Ireland	2008	409	36.1 ± 54.70	84
Levi	Israel	2009	199	21.1 ± 19.50	19
Fuchs	USA	2010	171	20.5 ± 17.50	14
Sauer	USA	2011	86	15.1 ± 18.00	6
Kroeker	Canada	2011	371	14.3 ± 1.45	27
Butcher	UK	2012	127	16.06 ± 17.52	8
Ciáurriz-Munuce	Spain	2012	235	33.4 ± 32.10	49
Hou	USA	2013	146	13.35 ± 34.71	26
Chatu	UK	2013	217	7.2 ± 14.59	29
Jung	Korea	2013	777	53.6 ± 66.40	270
Estay	Chile	2015	82	29.9 ± 41.54	16
Magro	Portugal	2015	451	-	72
Englund	Sweden	2016	103	-	20
Bousorra	Tunisia	2016	167	18.8 ± 18.90	-
Naidu	Malaysia	2017	36	18.58 ± 33.35	-
Rodríguez	Spain	2017	267	20.8 ± 31.40	41
Langevin	Canada	2019	157	27.5 ± 49.50	-
Nguyen	Canada	2019	28,797	25.98 ± 39.28	4491

**Table 2:** Characteristics of radiation exposure in UC patients

Author	Country	Year	Numbers	Radiation exposure (mSv)	High radiation exposure(*n*)
NewhamE	Australia	2007	37	-	2
Peloquin	USA	2008	112	10.50 ± 62.75	-
Levi	Israel	2009	125	15.10 ± 20.40	4
Fuchs	USA	2010	86	11.70 ± 9.90	1
Sauer	USA	2011	31	7.20 ± 8.50	0
Kroeker	Canada	2011	182	5.90 ± 0.81	12
Butcher	UK	2012	144	4.89 ± 7.47	0
Hou	USA	2013	126	7.40 ± 27.29	11
Chatu	UK	2013	198	2.80 ± 6.00	3
Jung	Korea	2013	1422	16.40 ± 29.20	119
Estay	Chile	2015	243	5.92 ± 15.13	6
Englund	Sweden	2016	304	-	33
Naidu	Malaysia	2017	76	3.65 ± 8.36	-
Langevin	Canada	2019	41	6.80 ± 14.80	-
Nguyen	Canada	2019	31,727	11.98 ± 27.81	1956

Furthermore, we compared whether there was a difference in radiation exposure level before and after 2012 in order to judge whether the increase in imaging methods in recent years has affected radiation exposure. 11 articles on CD were published after 2012, while 6 articles were published before 2012 (Table 1). The pooled radiation exposure level was 26.42 ± 39.61 mSv after 2012 and 23.76 ± 38.46 mSv before 2012, and there was a statistical difference between two groups (*P* = 0.016). In addition, high radiation exposure rate was 16.10% after 2012 and 12.25% before 2012, and it also had statistical difference (*P* < 0.01). However, UC did not show similar results. 8 articles were published after 2012, while 5 articles were published before 2012 (Table 2). The pooled radiation exposure level was 11.99 ± 27.66 mSv after 2012 and 10.01 ± 30.76 mSv before 2012, and high radiation exposure rate was 6.64% after 2012 and 4.30% before 2012. Neither parameter had statistical difference (*P* = 0.1 and *P* = 0.62, respectively).

**Fig. 4:**
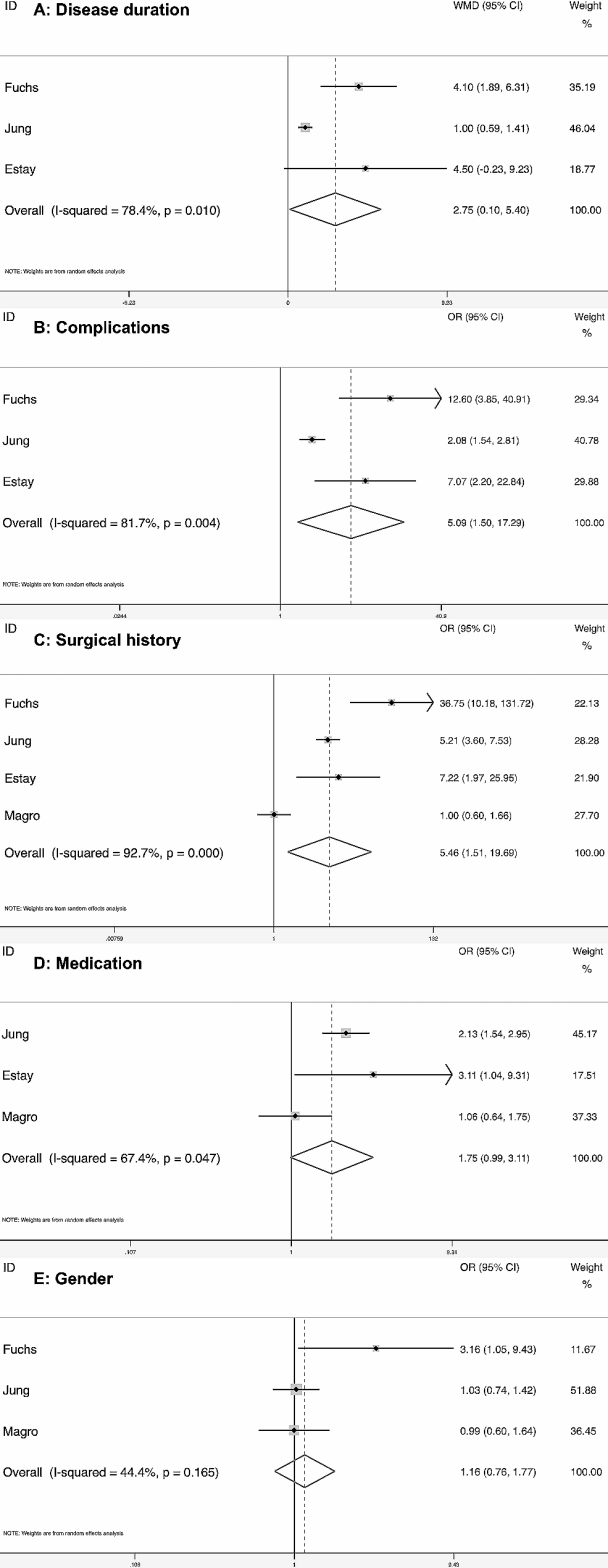
Forest plot showed odds ratio of risk factors of high radiation exposure grouped according to exposure

Finally, the study analyzed factors affecting radiation exposure. Due to lack of data, we only analyzed the influencing factors of CD. Disease duration, gender, complications, surgical history, and medication were the factors for our analysis. Through subgroup analysis, it was found that disease duration (WMD = 2.75, 95% CI = [0.10, 5.40] mSv), complications (OR = 5.09, 95% CI = [1.50, 17.29]), and surgical history (OR = 5.46, 95% CI = [1.51, 19.69]) significantly increased the proportion of high radiation exposure, while gender (OR = 1.16, 95% CI = [0.76, 1.77]) and medication (OR = 1.75, 95% CI = [0.99, 3.11]) had no effect. (Fig. 4)

Funnel plot analyses of studies assessing radiation exposure revealed no significant publication bias (*P* > 0.05). Sensitivity analysis showed that although some results were fluctuant, the overall results were stable and reliable.

## Discussion

This updated meta-analysis showed that radiation exposure of IBD patients was significantly increased, and the proportion of patients with high radiation exposure was also significantly increased. In addition, radiation exposure level of CD patients was significantly higher than that of UC patients, and the high radiation exposure of CD was related to disease duration, complications and surgical history.

Radiation exposure in IBD patients was significantly higher, which was depended on the course of diagnosis and treatment of the disease. Especially for CD patients, because the entire digestive tract may be involved, doctors need to conduct a comprehensive evaluation, especially the evaluation of the small intestine, which requires the use of small intestine CT and abdominal CT. It reported that incidence and mortality of solid cancer were positively associated with higher radiation dose and younger age of exposure [16]. And it has been reported that ionising radiation levels as low as 50 mSv have been contributed to the development of solid tumors [9]. Based on the results of this study and the characteristics of IBD patients with young age of onset and high radiation exposure [17], we believed that IBD patients may be exposed to an environment with a higher tumor incidence. So what can be done to reduce the risk of solid tumors in patients with IBD? First, we could propose the creation of an IBD patient radiation diary to record total radiation exposure and increase physician awareness of patient exposure to ionizing radiation [18]. Second, in tertiary care institutions, the frequency of magnetic resonance enterography (MRE) examinations can be increased to replace CT enterography (CTE). MRE is used to obtain cross-sectional imaging of small bowel without exposure to DMR, which can show the inflammation and fibrotic bowel wall in detail [19]. In a prospective study, Fiorino et al. found that MRE and CTE were similar accuracy in localizing CD, bowel wall enhancement, enteroenteric fistula, and MRE was superior to CTE for assessing strictures and bowel wall thickening [20]. Therefore, European Crohn’s and Colitis Organisation advocate increased routine usage of MRI for the assessment of small bowel CD [21]. According to the results of this study, why do the articles published in recent years showed that radiation exposure dose of IBD was higher than before. The authors believed that there were many reasons for this. First, the popularity of MRE is still only available in large general hospitals. Therefore, CTE remains the primary usage of IBD examination. Second, with the tense medical environment, doctors are more careful to deal with complications that may occur at any time during the diagnosis and treatment of patients and pay more attention to the efficacy of patients, so the frequency of examinations may be increased. Finally, Although the article was published in recent years, the patients included in the article may go back several years.

The results of this study showed that disease duration, complications and surgical history were associated with high radiation exposure, which was clearly closely related to the diagnosis and follow-up of the disease. The earlier the onset, the earlier the initial exposure. In addition, complications and surgical history have also added additional imaging tests to assess the severity of the disease. CT imaging offers advantages of rapid acquisition of images, high sensitivity, widespread availability, and specificity for the detection of intestinal and extra-intestinal disease [22]. Combined with the improved visualization of the small bowel mucosa by CTE, the assessment of small bowel disease activity is more accurate [23]. However, previous studies have shown that the role of CT in assessing intestinal disease activity may be limited [24]. In turn, radiation-induced cancer occurs in 1/1000 patients who undergo at least10 mSv CT scan [25]. Therefore, the appropriate imaging examination methods and frequency in the process of IBD diagnosis and treatment still require doctors to pay close attention.

On the basis of previous studies, this study has carried out a more detailed and systematic study and obtained more convincing results, but there were still some shortcomings needed to be pointed out. First, this study included data from multiple centers, which can lead to patient heterogeneity. Although sensitivity analysis showed the overall results were stable and reliable, the existence of heterogeneity still made this study only select random effect model for data analysis. The inconsistency of equipment models in different centers, the inconsistency of doctors’ cognition of diseases, and the compliance of patients would all affect the total radiation exposure. Moreover, this study has conducted extensive screening of papers. But based on the data provided by the published papers, the data of some included papers was not complete. We also asked the authors about the data through email, but unfortunately there was no reply. Additionally, the estimated radiation dose may be greater or less than the actual exposure. It is also possible that tests performed at other centers may not have been captured, leading to underestimate the total radiation dose. Second, we lacked studies with large sample data. Some studies included limited patients, which affected the reliability of the results. In particular, in the subgroup analysis of high exposure risk factors, the number of articles and patients included was limited, so the reliability of the results was limited. Finally, we lacked longer-term follow-up and prospective studies to analyze the risk of solid tumor development in high radiation exposure patients. The emergence of such results will have important guiding significance for the selection of imaging examinations in the process of IBD diagnosis and treatment.

## Conclusion

In conclusion, this study found that radiation exposure level of IBD patients was high, and exposure level of CD patients was higher than UC, which revealed the radiation risk in the process of diagnosis and treatment of IBD patients. In the future, longer follow-up and prospective studies are needed to reveal the relationship between high radiation exposure and solid tumorigenesis.

### Electronic supplementary material

Below is the link to the electronic supplementary material.

Supplementary Material 1

## Data Availability

The datasets used and/or analysed during the current study are available from the corresponding author on reasonable request.

## Abbreviations

IBD: Inflammatory bowel disease
CD: Crohn’s disease
UC: Ulcerative colitis
DMR: Diagnostic medical radiation
mSv: Millisieverts
OR: Odds ratio
CI: Confidence interval
MRE: Magnetic resonance enterography
CTE: CT enterography
